# Cold-Inducible RNA Binding Protein Impedes Breast Tumor Growth in the PyMT Murine Model for Breast Cancer

**DOI:** 10.3390/biomedicines12020340

**Published:** 2024-02-01

**Authors:** Daniel A. Lujan, Joey L. Ochoa, Ellen J. Beswick, Tamara A. Howard, Helen J. Hathaway, Nora I. Perrone-Bizzozero, Rebecca S. Hartley

**Affiliations:** 1 Department of Cell Biology and Physiology, University of New Mexico School of Medicine, Albuquerque, NM 87131, USA; dlujan7189@gmail.com (D.A.L.); lyric.joey@gmail.com (J.L.O.); thoward@salud.unm.edu (T.A.H.); hhathaway@salud.unm.edu (H.J.H.); 2 Department of Internal Medicine, University of Kentucky College of Medicine, Lexington, KY 40506, USA; ellen.beswick@uky.edu; 3 Department of Neurosciences, University of New Mexico School of Medicine, Albuquerque, NM 87131, USA; nbizzozero@salud.unm.edu

**Keywords:** CIRP, PyMT, Py2T, breast cancer, inflammation

## Abstract

RNA binding proteins (RBPs) post-transcriptionally regulate gene expression by associating with regulatory sequences in the untranslated regions of mRNAs. Cold-inducible RBP (CIRP) is a stress-induced RBP that was recently shown to modulate inflammation in response to cellular stress, where it increases or decreases pro-tumorigenic (proinflammatory) cytokines in different contexts. CIRP expression is altered in several cancers, including breast cancer, but the effects of CIRP on inflammation in breast cancer is not known. Here, we investigate if CIRP alters growth and the inflammatory profile of breast tumors. Transgenic mice overexpressing CIRP in the mammary epithelium were crossed with the PyMT mouse model of breast cancer, and the effects on both early and late tumorigenesis and inflammation were assessed. The effects of CIRP knockdown were also assessed in Py2T cell grafts. Overexpression of CIRP led to decreased tumorigenesis in the PyMT mouse model. Conversely, the knockdown of CIRP in Py2T cell grafts led to increased tumor growth. Luminex cytokine assays assessed the effects on the inflammatory environment. CIRP/PyMT mammary glands/mammary tumors and serum had decreased cytokines that promote inflammation, angiogenesis, and metastasis compared to PyMT mammary glands and serum, documenting a shift towards an environment less supportive of tumorigenesis. CIRP overexpression also decreased CD4^+^ helper T cells and increased CD8^+^ cytotoxic T cells in mammary tumors. Overall, these data support a role for CIRP as a potent antitumor molecule that suppresses both local and systemic pro-tumorigenic inflammation.

## 1. Introduction

RNA binding proteins (RBPs) play key roles in RNA dynamics, including subcellular localization, translational efficiency, and metabolism [1,2]. Given the numerous physiological roles of RBPs, they are also key molecules in disease, including cancer [3,4,5,6,7,8,9]. Cold-inducible RNA binding protein (CIRP), also known as hnRNPA18 and CIRBP, is a glycine-rich RBP that contains an RNA-recognition motif (RRM) and a carboxyl-terminal domain with several RGG motifs that facilitate protein–protein interactions [10,11]. CIRP is expressed in a wide variety of tissues and is induced in response to various cellular stresses, where it translocates from the nucleus to the cytosol and binds target mRNAs [12,13,14]. Recent studies have detailed the role of CIRP in modulating inflammation in a variety of contexts, including several types of cancer [15,16,17,18,19,20,21,22,23,24,25]. CIRP acts as tumor suppressor in some cases [26,27,28,29,30,31,32] but also promotes tumor growth [33,34,35,36,37,38,39]. Collectively, the role CIRP plays in inflammation and cancer is likely context dependent. CIRP was previously shown to be a positive regulator of cyclin E1, HIF-1α, and cytostatin C in breast cancer [40,41,42]. We have previously studied the effects of CIRP overexpression in normal mammary gland development [28]. However, CIRP’s function in breast cancer has largely been based on in vitro studies. Here, we show that CIRP decreases tumor growth and metastasis in vivo while decreasing pro-tumorigenic inflammation.

## 2. Materials and Methods

### 2.1. Transgenic Mice

MMTV-PyMT (FVB/N-Tg (MMTV-PyMT) 634 Mul/J) mice were purchased from the Jackson Laboratory (Bar Harbor, ME), and a breeding colony was maintained at the University of New Mexico Health Sciences Center Animal Research Facility (ARF). Mice overexpressing the full-length human coding sequence of CIRP (FVB/N-Tg MMTV-hCIRP) were generated and characterized as previously described [28]. The animals were housed at the ARF in a temperature-controlled environment (23 °C) with a 12 h light–12 h dark cycle. Hemizygous CIRP females (CIRP/+) were crossed with hemizygous PyMT males (PyMT/+) to generate CIRP/PyMT (PyMT/+, CIRP/+) female mice. Mice were genotyped using DNA extracted from a 0.2 cm tail clipping taken at weaning. Genotyping qPCR reactions were carried out using the Applied Biosystems Fast PCR 7500 system (Applied Biosystems, Foster City, CA, USA). Then, 10 μL reactions were used consisting of 5 μL Fast-SYBR Green (Applied Biosystems, Foster City, CA, USA), 1 μL of mixed 20 μM forward and reverse genotyping primers, 3 μL of RNase-free water, and 1 μL of template DNA. The genotyping primers are listed in Table S1. For all the mouse studies described below, CIRP/PyMT mice were compared to PyMT mice.

### 2.2. Collection of Tissue

Mouse tumors were dissected from PyMT or CIRP/PyMT mice at either 7 weeks or 14 weeks of age. Mammary glands, mammary tumors, and lungs were collected and fixed in either 4% paraformaldehyde or 10% buffered formalin for 24–48 h. Serum was also collected for the cytokine analyses. One number 4 mammary gland was cut in half lengthwise, with one half homogenized in Trizol (Invitrogen, Carlsbad, CA, USA) and frozen at −80 °C for subsequent RNA extraction and one half frozen at −80 °C for subsequent protein extraction. Tissue supernatants were obtained by incubating a 6–10 mg portion of mammary tumor or mammary gland (containing epithelial ducts from the proximal end of the mammary gland) in 500 μL of complete tissue culture media overnight (16 h) at 37 °C with 5% CO_2_. Supernatants were then used in either Luminex cytokine arrays or in ELISA kits to quantify the CIRP protein levels. All other tissues were dehydrated through ascending concentrations of ethanol, cleared with Hemo-De, and embedded in Paraplast Plus. For mice with syngeneic Py2T cell grafts, only mammary tumors were harvested at 14 weeks of age for their final weight.

### 2.3. Whole Mounts

Whole mounts of number 4 mammary glands from 7-week-old mice were prepared by rinsing paraformaldehyde-fixed mammary glands two times with acetone over an 8–24 h period, followed by a series of water rinses over 1 h, and staining with carmine alum (0.2% carmine red and 0.5% aluminum potassium sulfate) overnight with gentle shaking [43]. The glands were destained in a graded series of ethanol and stored in methyl salicylate. All steps were performed at room temperature. The whole mounts were imaged using a stereomicroscope and Motic camera with Motic Images Plus 2.0 ML software. Multiple images of the mammary gland were taken, and composite images of the whole mounts were created using Adobe Photoshop. For the whole mounts of mammary glands from 7-week-old mice (early tumorigenesis), mammary intraepithelial neoplasias (MINs) within these glands were quantitated using ImageJ software (Version 1.46r). A grid of 10 mm squares was superimposed onto the whole mounts, with the squares first being counted for those that contained mammary epithelial tissue and then those containing mammary epithelial tissue and neoplasia. A percentage of MIN burden was calculated by dividing the MIN count by the total mammary epithelial tissue count and multiplying by 100.

### 2.4. Histology and Immunostaining

Formalin-fixed paraffin-embedded (FFPE) mouse mammary glands and lungs were sectioned in 50 μm levels for 20 levels, with three slides taken per level. Sections were 5 μm in thickness. One slide from each level was stained with hematoxylin and eosin (H&E) for histological examination, and one was used for immunohistochemistry (IHC). For the histological examination, sections were stained in Harris’ hematoxylin (Sigma-Aldrich, Waltham, MA, USA) for 30–45 s, followed by 1–10 dips in acid alcohol and a 45 s dip in Eosin Y. Sections were then dehydrated through two-minute dips in ascending concentrations of ethanol. Coverslips were mounted on slides using DPX mounting medium (Electron Microscopy Services, Hatfield, PA, USA), and the slides were analyzed for histopathology. For the assessment of 7-week-old mammary glands, five 60× fields in the central portion of the most advanced lesion were assessed. Tumors (14 weeks) and glands containing MINs (7 weeks) were both sectioned and processed as described above. Lung sections were also analyzed to determine the number of metastatic foci. Only lungs taken from mice at 14 weeks of age were sectioned to assess the pulmonary metastases. The lungs were H&E-stained using the same method described above.

For immunostaining, the paraffin sections were rehydrated to 1× PBS (IFA) or 1× PBS with 0.1% Tween 20 (PBST) and subjected to antigen retrieval for 15–20 min at 90–95 °C in a water bath in 10 mM Tris pH 9.0, 1 mM EDTA, and 0.05% Tween 20. Endogenous peroxidase activity was suppressed by incubation with 3% H_2_O_2_ in PBS for 30 min with agitation, followed by 1× PBS + 3% normal goat serum (NGS) + 0.1% Triton X-100 for 30 min to prevent nonspecific staining. Sections were incubated in Ki67 primary antibody (as indicated in Table S2) diluted in 1× PBS + 3% NGS + 0.1% Tween 20 overnight at 4 °C. Following the primary antibody, sections were washed 3 times in 1x PBST for 10 min each and then incubated in donkey anti-rabbit IgG HRP secondary antibody for 1 h at room temperature. Sections were developed using DAB as chromogen and washed in 1× PBS twice for 5 min, followed by a wash with diH_2_O. Sections were then counterstained in Harris’ progressive hematoxylin for 15 s, dehydrated, and mounted using DPX mount (Electron Microscopy Science, Hatfield, PA, USA) and a coverslip. Slides were viewed and photographed using a Nikon Eclipse e400 microscope equipped with a Nikon DS-Fi1 camera (El Segundo, CA, USA) and analyzed using ImageJ software (1.46r). For Ki67 staining, bar graphs were produced using representative images to count the HRP-stained nuclei against non-HRP-stained nuclei in mammary glands from PyMT and CIRP/PyMT mice.

### 2.5. Syngeneic Py2T Tumor Cell Grafts

Py2T cells were generously provided by co-author Dr. Helen Hathaway. The cells were originally derived from a primary mammary tumor from a transgenic MMTV-PyMT mouse on an FVB background. The cells were characterized using the method described by Waldmeier et al. [44]. The cells were maintained in DMEM/F-12 media with 5% fetal bovine serum and 1% penicillin/streptomycin and cultured at 37 °C and 5% CO_2_. The cells were used between passages 3 and 12.

Control Py2T cells or CIRP knockdown Py2T cells (1 × 10^6^) were injected into the number 4 mammary fat pads of 7-week-old wild-type FVB mice. One fat pad received control Py2T cells, and the other in the same mouse received CIRP knockdown Py2T cells. Tumors were measured using digital calipers twice per week for 7 weeks, and the mice were euthanized and tumors harvested at 14 weeks of age. Tumors were weighed at necropsy (7 weeks post-injection). The final tumor weight was averaged and plotted for each genotype (n = 5). Digital caliper measurements for length and width were used to calculate the tumor volume using the tumor ellipsoid formula (π/6 × length × width) [45]. The tumor volume was plotted over time as age in weeks.

### 2.6. CIRP Knockdown

Stable CIRP knockdown was achieved using the Alt-R CRISPR/Cas9 system (IDT). Guide RNAs (Alt-R crRNAs) were designed using the design tool by IDT to target the coding sequence of the murine CIRP gene. The sequences of each Alt-R crRNA are shown in Table S3. crRNA-trans-activating crRNA (tracrRNA) duplexes were prepared by mixing equimolar concentrations of each crRNA and tracrRNA (10 μl Alt-R crRNA and 10 μL Alt-R tracrRNA) in a nuclease-free duplex butter (IDT catalog #11010301) and annealing at 95 °C for 5 min in a thermocycler. Equal amounts (working concentration of 1 μM) of each Alt-R crRNA-tracrRNA duplex (3 total) were then used to prepare crRNA-tracrRNA-Cas9 RNP complexes. Alt-R S.p. HiFi Cas9 nuclease (IDT catalog #1081060) was mixed at a recommended working concentration of 1 μM, along with 1 μM of mixed crRNA-tracrRNA duplexes, into Opti-MEM media (Thermo Fisher Catalog #51985091). RNP complexes were mixed with gentle pipetting or gentle vortexing and incubated at room temperature for 20–30 min. RNP complexes were used to prepare transfection complexes by mixing 750 μL of RNP complex with 36 μL of CRISPRMAX Cas9 lipofectamine reagent (Thermo Fisher Catalog #CMAX00001) and 714 μL of Opti-MEM media. Transfection complexes were mixed by gentle pipetting and incubating at room temperature for 20–30 min. Transfection complexes were then gently mixed with Py2T cells at a concentration of 400,000 cells/mL and plated in 6-well plates. The cells were mixed with either the positive control (targeting the HPRT gene) crRNA RNP complex, negative control crRNA RNP complex, CIRP crRNA RNP complex, or lipofectamine only (untreated). The cells were allowed to grow for 48 h before splitting and harvesting some for the protein and preserving the rest for subsequent transfections. A total of three transfections were performed to achieve the desired knockout/knockdown efficiency of ~85% at the protein level.

The CIRP knockdown efficiency was measured at the protein level using an ELISA specific for the murine CIRP protein (CUSABIO, Wuhan, China). The cells were trypsinized and collected from 6-well plates after 48 h of incubation with RNP complexes. Half of the cells were kept for subsequent transfections, and half were harvested for the protein for use in the ELISA. The CIRP knockdown efficiency is shown in Figure S1.

### 2.7. Tissue Supernatants and Luminex Arrays

Tissue supernatants were obtained by incubating a 6–10 mg piece of mammary tumor tissue in DMEM/F12 cell culture media with 5% fetal bovine serum overnight. The resulting tissue supernatants and serum were used to assess 25 different cytokine protein levels (G-CSF, GM-CSF, IFN-γ, IL-1α, IL-1β, IL-2, IL-3, IL-4, IL-5, IL-6, IL-7, IL-9, IL-10, IL-12 (p40), IL-12 (p70), IL-13, IL-15, IL-17, IP-10, KC, LIF, LIX, MCP-1, M-CSF, MIG, MIP-1α, MIP-1β, MIP-2, RANTES, TNF-α, VEGF-A, and Eotaxin/CCL11). Cytokine production in mammary tissue was assessed using Milliplex mouse cytokine array assays (EMD Millipore, Billerica, MA, USA). Multiplex arrays were used for these analyses in accordance with the manufacturer’s protocols. The results for all the cytokines are shown in Tables S4 and S5 for the tumors and serum from 14-week-old mice.

### 2.8. CIRP ELISA

Human CIRP (hCIRP) and mouse CIRP (mCIRP)-specific enzyme-linked immunosorbent assay (ELISA) kits were from CUSABIO (Wuhan, China). The serum samples were diluted 10-fold, in accordance with the manufacturer’s instructions. Tissue homogenates were prepared from 100 mg of mammary tumor tissues using 2 freeze–thaw cycles followed by 5 min of centrifugation at 5000× *g* at 2–8 °C. The resulting supernatants were collected and assayed in the ELISA protein assays in accordance with the manufacturer’s instructions. These colorimetric assays were read on a Tecan Infinite F500 (Tecan, Männedorf, Switzerland) microplate reader at 450 nm.

### 2.9. Murine Breast Tumor Digestion

Mammary tumors developed in PyMT/+ and CIRP/PyMT mice were isolated from the mice at necropsy (14 weeks of age). The tumors were washed in PBS, cut into 6–8 roughly equal pieces, and placed in a 10 mL digestion solution of DMEM/F12 + 5% fetal bovine serum with 1× collagenase/hyaluronidase in DMEM (Stemcell Technologies Cambridge, MA, USA), 0.05 U/mL Dispase (Stemcell Technologies Cambridge, MA, USA), and DNase I Solution (Stemcell Technologies Cambridge, MA, USA). The tumors were incubated at 37 °C for 100 min and briefly vortexed at 60% power every 10 min. The resulting cell suspension was then vortexed at full power for 30–45 s and then strained through a 70 μm cell strainer. The cells were then pelleted and washed in PBS twice. The cells were then resuspended in flow cytometry staining buffer containing PBS with 2 mM EDTA and 5% FBS.

### 2.10. Cell Staining and Flow Cytometry

Prior to the addition of antibodies, the cells were suspended in staining buffer containing phosphate-buffered saline and 2 mM EDTA with 5% fetal bovine serum. The cells were then incubated with 8 antibodies specific for F4/80, CD11b, Ly6C, Ly6G, CD19, CD3, CD4, and CD8 (See Table S2 for the working concentrations) for 30 min at 4 °C. Two wash steps were then completed, where the cells were pelleted by spinning at 500× *g* for 5 min at 4 °C and washed in PBS. The cells were resuspended in staining buffer prior to assaying on the Attune NxT flow cytometer (Thermo Fisher Scientific, Waltham, MA, USA). The cells were first gated to eliminate dead cells and then to identify only singlets before gating for specific immune cells markers. A flow cytometry panel was designed using the Fluorofinder Design Tool (Broomfield, CO, USA) to minimize the spillover from fluorochromes.

### 2.11. Statistics

The average tumor weight, tumor burden, average tumor volume, levels of individual cytokines, average number of metastatic foci, and average percentage gated were compared with two sample *t*-tests for comparisons of two groups (PyMT/+ vs. PyMT/CIRP). *p*-values < 0.05 were considered significant. For the cytokine analyses, *p*-values were corrected using the Benjamini–Hochberg method to control for the false discovery rate (FDR). A correlation analysis was done to obtain the R^2^ value. Statistical analyses were performed using Microsoft Excel for Mac version 16.79.2. Experiments were repeated a minimum of 3 times, with the numbers of samples indicated in each figure.

## 3. Results

### 3.1. Human CIRP Expression Impeded Early Tumorigenesis in MMTV-PyMT Mice

**Figure 1:**
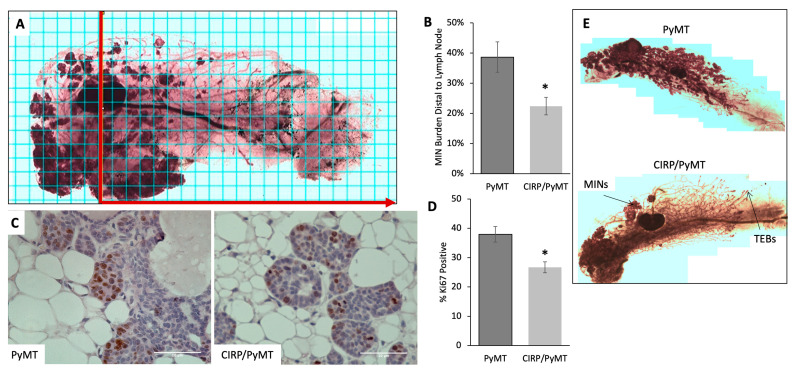
CIRP decreases early tumorigenesis and proliferation in 7-week-old PyMT mice. (**A**) Representative image of a mammary gland whole mount from a 7-week-old PyMT mouse with the grid overlay and division of the lymph node (red line) used for quantitation of the MIN burden in panel B. (**B**) Quantitation of mammary intraepithelial neoplasias (MINs). The MIN burden is the percentage of the gland containing MINs distal to the lymph node (n = 8). (**C**) Representative Ki67 immunostaining (brown) of PyMT/+ and CIRP/PyMT mammary glands. Five-micron sections were counterstained with hematoxylin (purple). (**D**) Quantitation of Ki67-positive cells relative to the total cells (n = 4). * *p* < 0.05, two-sample *t*-test. (**E**) Representative PyMT and CIRP/PyMT mammary gland whole mounts. MINs and terminal end buds (TEBs) are labeled.

To assess the effects of CIRP on early mammary tumor development, mammary gland whole mounts were prepared from the number 4 mammary gland from 7-week-old MMTV-PyMT and MMTV-CIRP/PyMT mice. ImageJ [46] was used to assess the total area of mammary intraepithelial neoplasias (MINs) relative to the total epithelial area. Quantitation revealed that PyMT mice had significantly more MINs than CIRP/PyMT mice. Since MINs develop in a proximal-to-distal direction, as the epithelial ducts proliferate and extend into the fat pad during mammary gland development [47,48,49], the MIN burden distal to the midline of the lymph node was also assessed. Figure 1A shows a representative whole mount mammary gland image for a CIRP/PyMT mouse overlaid with a counting grid. The area counted is indicated by a red line drawn vertically through the middle of the lymph node and extending horizontally in the direction of the arrow. Figure 1B shows that CIRP/PyMT mice had significantly fewer MINs distal to the lymph node when compared to PyMT mice (23% vs. 39%, respectively).

Histopathological analysis of the mammary glands of 7-week-old mice confirmed that not only were there fewer MINs in the CIRP/PyMT mammary glands but that the lesions were less advanced. The mammary lesions in both mice were similar in character, with a primary lesion containing multiple MINs and occasional foci suggestive of early carcinoma. However, the mammary lesions in CIRP/PyMT mice were less advanced than those found in PyMT mice, with PyMT mice having larger primary lesions, greater secondary duct involvement, and more advanced secondary lesions (Table 1). The mammary lesions from PyMT mice also had more mitotic figures and fewer apoptotic figures on average when compared to the lesions in CIRP/PyMT mice (Table 1). These results suggested that an altered balance between proliferation and apoptosis could be responsible for the less advanced lesions and reduced number of MINs in CIRP/PyMT mice.

Based on the results shown in Figure 1B and Table 1, the effect of CIRP overexpression on epithelial proliferation was examined. Proliferation was assessed by immunostaining for proliferation marker Ki67. Figure 1C shows representative Ki67 staining (brown) in sections of PyMT and CIRP/PyMT mammary glands. Figure 1D shows that there was significantly less proliferation in CIRP/PyMT mammary glands (24% of epithelial cells were Ki67-positive) compared to the mammary glands from PyMT mice (38% of epithelial cells were Ki67-positive). This finding indicated that CIRP reduced epithelial proliferation, which may account, in part, for the reduced MIN burden and less advanced lesions in CIRP/PyMT mice. Figure 1E shows the visual difference in MINs between mammary gland whole mounts taken from PyMT and CIRP/PyMT mice at 7 weeks of age.

### 3.2. CIRP Impeded Late Tumorigenesis and Pulmonary Metatasis

**Figure 2:**
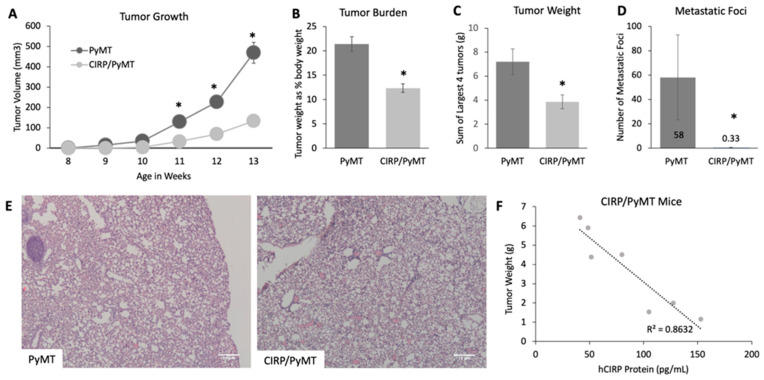
CIRP decreases late tumorigenesis and pulmonary metastasis in 14-week-old PyMT mice. (**A**) Tumor volume as calculated from the ellipsoid area formula V = π/6 × length × width (n = 13). (**B**) Tumor burden is the tumor weight as a percentage of the total body weight (n = 13). (**C**) Sum of the largest four tumors averaged from mice of each genotype (n = 13). (**D**) Average number of pulmonary metastatic foci from each genotype (n = 5). (**E**) Representative images of pulmonary metastatic foci taken at 4× magnification. (**F**) Correlation analysis between the hCIRP protein level (measured by ELISA) and tumor size (sum of the 4 largest tumors); R^2^ = 0.8632. * *p*-value < 0.05.

Tumor progression was tracked in mice from 8 to 14 weeks of age in order to assess the effects on later tumorigenesis. An analysis of the tumor volume revealed that the tumors in CIRP/PyMT mice were smaller and grew more slowly when compared to PyMT mice at the same time point, with significant differences in the total tumor volume at 11, 12, and 13 weeks of age (Figure 2A). At 14 weeks of age, the mice were weighed at necropsy before the tumors were removed and weighed to determine the tumor burden. Figure 2B shows that CIRP/PyMT mice had a significantly lower tumor burden when compared to PyMT mice. The weights of the heaviest four tumors were also measured (Figure 2C), with CIRP/PyMT mice having smaller tumors when compared to the tumors from PyMT mice. A large percentage of PyMT mice developed not only carcinomas by 14 weeks of age but also pulmonary metastases [45]. To assess CIRP’s effect on pulmonary metastases, the lungs from PyMT and CIRP/PyMT mice were sectioned and stained with hematoxylin and eosin and imaged to quantify the number of metastatic foci. Figure 2D shows the average number of metastatic foci in the lungs from CIRP/PyMT and PyMT mice. The lungs from CIRP/PyMT mice had greatly reduced pulmonary lesions when compared to PyMT mice, an average of 0.3 compared to 58. Taken together, these results suggested that CIRP impeded late stage tumor growth and metastasis.

To more directly assess the relationship between CIRP expression and tumor development, ELISAs were used to specifically quantitate the human CIRP protein in mammary tumors from 14-week-old CIRP/PyMT mice. A correlation analysis was then used to assess the relationship between the CIRP level and tumor weight. Figure 2E shows the results of this analysis. As would be expected if CIRP were inhibiting tumor growth, CIRP expression negatively correlated with the tumor size, and the tumor size decreased with the increased CIRP protein. These results showed that CIRP impedes breast tumorigenesis.

### 3.3. CIRP Knockdown Increased Tumor Growth in Py2T Tumor Cell Grafts

**Figure 3:**
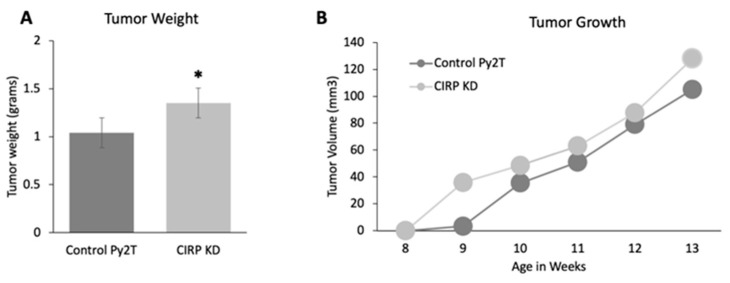
CIRP knockdown increases tumor growth in Py2T cell grafts. (**A**) Tumor weight shown at necropsy (n = 5). (**B**) Tumor volume in mm^3^ from the ellipsoid area formula V = π/6 × length × width (n = 5), as measured at the indicated times. * *p*-value < 0.05 (two sample *t*-test).

The effects of CIRP loss on tumor growth was also assessed. Py2T cells were generated by passaging the primary mammary tumor cells from a transgenic PyMT mouse and characterized as described [44]. CIRP was stably knocked down in these cells using CRISPR-Cas9. Figure S1 shows that the CIRP protein was decreased by at least 85%, down to 22 pg/mL (at the minimum level of detection according to the sensitivity of the ELISA), as compared to an average of 140 pg/mL in the control cells. Control-treated Py2T or CIRP knockdown (CIRP KD) Py2T cells were injected into the number 4 mammary gland of wild-type FVB mice, and tumor growth was monitored. Figure 3A shows that CIRP KD cell grafts resulted in larger tumors when compared to control Py2T cell grafts at 14 weeks (weighed at necropsy). Figure 3B shows that, although there was no significant difference in the tumor growth rate overall, the tumor latency was decreased with CIRP KD cells when compared to control Py2T cells. Two weeks after injection of the cells, CIRP KD Py2T cells formed a tumor of an average volume of 36 mm^3^, while negative control-treated Py2T cells produced a just-detectable tumor of an average volume of 3 mm^3^. The overall increase in tumor size coupled with decreased latency in CIRP KD cells reinforced the overexpression data showing that CIRP suppressed tumorigenesis.

### 3.4. CIRP Decreases Pro-Tumorigenic Cytokines in Mammary Tumors

CIRP has been shown to modulate inflammation in various types of cancer [15,17,18,19,21]. In addition, the cytokine levels in both serum (systemic inflammation) and mammary glands (local inflammation) are known to influence tumor growth in both human breast cancer [50,51] and in the PyMT mouse model [47,52]. To ask if the effects seen on tumorigenesis were secondary to the changes in the inflammatory environment, cytokines produced by mammary glands (7 weeks) and mammary tumors (14 weeks) were assessed using tissue supernatants [53]. Tissue supernatants were obtained by incubating mammary tissue from 7-week-old or mammary tumors from 14-week-old PyMT and CIRP/PyMT mice overnight in complete cell culture media. Cytokines released into the media were assayed using multiplex Luminex cytokine arrays, as were cytokines in serum from these mice.

**Figure 4:**
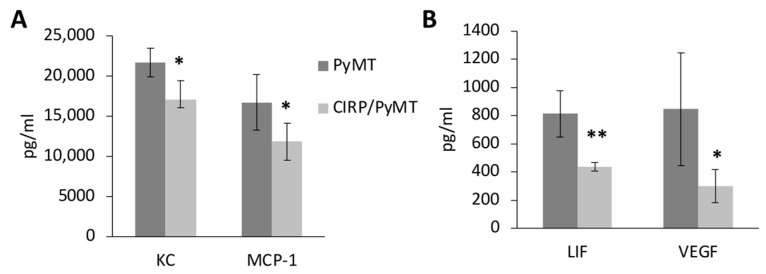
CIRP decreases pro-tumorigenic cytokines in early tumorigenesis in 7-week-old mice. Quantitation of cytokines in supernatants from overnight cultures of PyMT/+ and CIRP/PyMT mammary glands, measured by multiplex ELISA (n = 3). (**A**) Levels of KC and MCP-1. (**B**) Levels of LIF and VEGF-A. * *p* < 0.05 and ** *p* < 0.005.

Of the 25 cytokines assessed, 7-week-old CIRP/PyMT mammary glands produced significantly lower levels of chemokine ligand 1 (KC), monocyte chemoattractant protein (MCP-1), leukemia inhibitory factor (LIF), and vascular endothelial growth factor (VEGF-A) compared to PyMT mammary glands (Figure 4). LIF promotes tumorigenesis and metastasis in breast cancer [54] and was shown to inhibit p53 in colorectal cancer [55]. KC (CXCl1) promotes inflammation and metastasis by recruiting myeloid cells in breast cancer, including neutrophils [56]. MCP-1 (monocyte chemoattractant or CCL2) targets monocyte migration into the tumor microenvironment, facilitating metastatic seeding by VEGF-A-mediated angiogenesis [57]. CCL2 also increased the stromal density and cancer susceptibility in a mouse breast cancer model [58]. The assessment of serum cytokines in 7-week-old mice revealed no significant differences in the cytokine levels between CIRP/PyMT and PyMT mice. These results suggested that CIRP impeded early mammary tumor growth by decreasing local pro-tumorigenic cytokine production.

**Figure 5:**
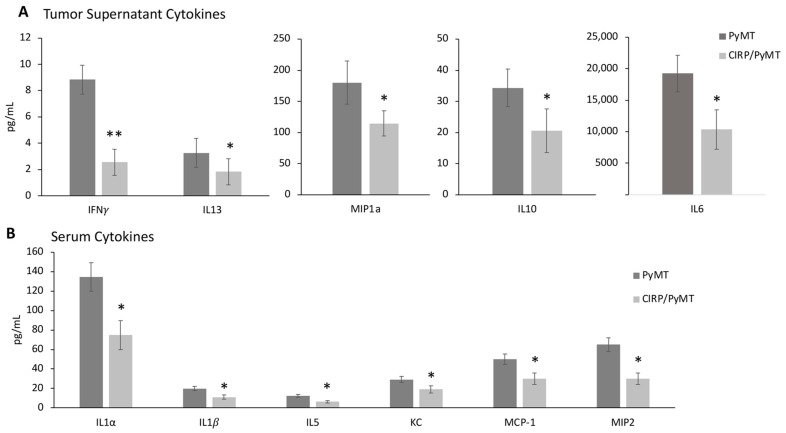
CIRP decreases pro-metastatic and pro-tumorigenic cytokines in late tumorigenesis in 14-week-old mice. (**A**) Quantitation of cytokines in supernatants from overnight cultures of PyMT/+ and CIRP/PyMT mammary gland tumors, measured by multiplex ELISA (n = 6). (**B**) Quantitation of cytokines in serum from 14-week-old PyMT/+ mice and CIRP/PyMT mice (n = 6). * *p* < 0.05 and ** *p* < 0.01 (two sample *t*-test). *p*-values were corrected using the Benjamini–Hochberg method (adjusted range of 0.009–0.049).

Consistent with the results in 7-week-old mammary glands, several pro-tumorigenic cytokines were also significantly lower in the supernatants from CIRP/PyMT mice during late stage tumorigenesis (14 weeks) when compared to PyMT mice. There were significantly lower levels of interferon-γ (IFN-γ), interleukin-13 (IL-13), chemokine ligand 3 (MIP-1α), interleukin-10 (IL-10), and interleukin 6 (IL-6) (Figure 5A). In addition, the serum from 14-week-old CIRP/PyMT mice had significantly less interleukin-1α (IL-1α), interleukin-1β (IL-1β), interleukin-5 (IL-5), KC, MCP-1, and chemokine ligand 2 (MIP-2) (Figure 5B). IFN-γ is typically associated with a tumor rejection immune response; however, a high expression is not always beneficial in breast cancer [59,60]. MIP-1α has been linked to metastasis and chemoresistance in breast cancer [61]. IL-10 and IL-6 are both known to promote breast tumor growth and are often central to breast tumorigenesis and metastasis [62,63,64,65,66,67]. IL-13 is upregulated in breast cancer [68]. These results suggested that CIRP is decreasing the number of cytokines that are known to promote tumor growth in mice at both 7 and 14 weeks of age, locally and systemically. Tables S4 and S5 show the levels of all the cytokines assessed in 14-week-old tissue supernatants and serum.

### 3.5. CIRP Decreases CD4+ Helper T Cells and Increases CD8+ Cytotoxic T Cells in Mammary Tumors during Late Stage Tumorigenesis

**Figure 6:**
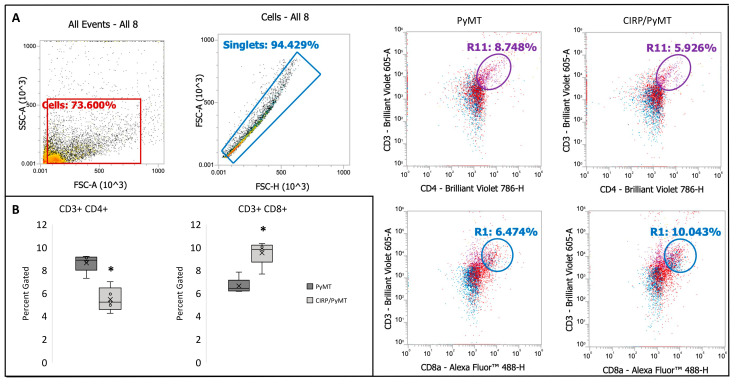
CIRP decreases CD4+ helper T cells and increases CD8+ cytotoxic T cells during late stage tumorigenesis in 14-week-old mice. (**A**) Representative dot plots showing the gating strategy for all events, for singlets, and for quantitating CD4+ (CD3+ and CD4+) and CD8+ (CD3+ and CD8+) T cells. Representative plots for tumor cells from a PyMT/+ mouse and a CIRP/PyMT mouse when gated for CD3 (*Y*-axis) and either CD4 or CD8 (*x*-axis) are shown. (**B**) Box plots show the average percent gated cells from the total cells in PyMT/+ and CIRP/PyMT mice. (n = 5), * *p* < 0.05.

In order to ask if the effects of CIRP on several pro-tumorigenic cytokines was secondary to affecting the immune cell cohort within the tumors, we performed flow cytometry to assess the levels of B cells (CD19+); CD4+ helper T cells (CD3+ and CD4+); CD8+ cytotoxic T cells (CD3+ and CD8+); macrophages (F4/80+); neutrophils (CD11b+, and Ly6G+, and Ly6C+); and monocytes (CD11b+ and Ly6G+). Analyses of these immune cells were chosen based on our results from the assessment of the cytokine levels. Mammary tumors were taken from PyMT/+ and CIRP/PyMT mice at 14 weeks of age and digested into a single-cell suspension. Of the five immune cell types analyzed, the percentages gated of macrophages, monocytes, neutrophils, and B cells were not significantly different between PyMT/+ mice and CIRP/PyMT mice. Interestingly, CIRP/PyMT tumors had significantly fewer CD4+ helper T cells and significantly more CD8+ cytotoxic T cells when compared to PyMT/+ mice (Figure 6). Higher levels of CD8+ cytotoxic T cells and lower levels of CD4+ helper T cells are typically associated with a better prognosis and outcome in human breast cancer [51,52,69]. These results suggest that the effects of CIRP on tumor growth and pro-tumorigenic cytokines is potentially due to changes in the immune cell cohort.

## 4. Discussion

Cold-inducible RBP (CIRP) is a stress-induced RBP that has been shown to modulate inflammation in response to cellular stress. CIRP expression is altered in several cancers, including breast cancer. While the normal physiological role of CIRP as a stress-induced RBP has been well characterized, more recent studies have detailed the roles for CIRP in several diseases, including different forms of cancer and in inflammation [15,16,17,18,19,20,21,22,23,24,25,26,27,28,29,32,34,37,38,40,41,42,70,71,72,73]. Our previous studies identified a role for CIRP in suppressing epithelial proliferation during mammary gland development, specifically at the transition from lactation to involution [28]. The current study assessed the potential effects of CIRP on the development and progression of mammary tumorigenesis in the MMTV-PyMT mouse model of breast cancer that recapitulates many key features of human disease.

To our knowledge, this is the first study to explore the role of CIRP in breast cancer and inflammation in vivo. Our results show that overexpression of human CIRP in the mammary epithelium impedes tumor growth and metastasis in the MMTV-PyMT model. Early tumorigenesis was inhibited, with fewer, less advanced lesions in CIRP/PyMT mammary glands when compared to PyMT glands, due in part to decreased proliferation. The inhibition of tumorigenesis extended into the late stages, with increased tumor latency, decreased tumor size and burden, and a remarkable decrease in pulmonary metastases. The final tumor size negatively correlated with the CIRP protein level, suggesting a direct influence of CIRP on tumor growth. These findings were reinforced by CIRP knockdown experiments. Py2T cells in which CIRP was stably knocked down had decreased tumor latency and larger tumor mass compared to control Py2T cells when grafted into wild-type FVB mammary glands. Consistent with these findings, other studies have demonstrated the role of CIRP in decreasing proliferation [10,26,27,70]. CIRP expression negatively correlated with malignancy in ovarian tumors, endometrial cancers, and nasal cancers [26,27,32,71,72]. A recent study identified CIRP as one of nine genes associated with the prognostic response to neoadjuvant therapy [73]. Our observations support the role for CIRP as a tumor suppressor that reduces the progression and invasive potential of mammary gland tumors and encourages further delineation of the effects and benefits of CIRP on breast (and potentially ovarian and endometrial) cancer development and progression.

Previous studies on the effects of CIRP on inflammation [74] support an investigation of how CIRP may affect the inflammatory environment within our models. Generally, our results showed that pro-tumorigenic cytokines were reduced in CIRP/PyMT mice when compared to PyMT mice at both early and late time points. Interestingly, an assessment of the cytokines produced by mammary glands with early lesions showed significantly lower levels of VEGF-A, whose role in angiogenesis in breast cancer is well established [75,76]. CIRP was also recently shown to negatively regulate angiogenesis in vivo [77]. However, there was no discernible difference in vascularity between the MINs examined by histopathology in 7-week-old CIRP/PyMT and PyMT mice. This discrepancy may be due to the early stage of the disease. Future studies will assess the vascularity and angiogenic markers such as VEGF and CD31 in mouse tissue and tumors by IHC to confirm or refute these results. The lower levels of cytokines LIF, MCP-1, and KC in the CIRP/PyMT supernatants likely played a role in the decreased tumor growth observed, as these cytokines are all known to maintain the pro-tumor microenvironment in breast cancer [54,55,56,57,58].

In late-stage tumorigenesis, the levels of pro-tumorigenic cytokines were decreased, both locally and systemically. The CIRP/PyMT mammary gland tumors showed decreased levels of IL-13, MIP-1α, IL-10, IL-1β, IL-5, IL-6, KC, MCP-1, MIP-2, LIF, and VEGF-A compared to PyMT tumors. In breast cancer, MIP-1α, MIP-2, KC, and MCP-1 are chemokines that recruit macrophages, neutrophils, and monocytes to maintain the tumor microenvironment [52]. MIP-1α and MIP-2 have also been linked to metastasis and chemoresistance in breast cancer [61]. IL-10 downregulates the expression of Th1-type cytokines and increases the immune tolerance of cancer in the tumor microenvironment [62,63,64], while IL-1β is associated with an increased risk for bone metastasis in human breast cancer [78]. LIF was shown to promote migration and metastasis in breast cancer through the AKT-mTOR pathway [54], and elevation of VEGF-A in breast cancer is typically associated with poor prognosis and higher rates of metastasis [75,76]. IL-6 is known to promote invasiveness through epithelial-to-mesenchymal transition and is associated with the formation and maintenance of breast cancer stem cells [67]. IL-5 and IL-13 have been reported as elevated in some breast cancers, with a potential role for IL-5 in enhancing the response to an immune checkpoint blockade [79,80].

In addition to the altered inflammatory cytokines, a decreased number of CD4+ helper T cells and an increased number CD8+ cytotoxic T cells were seen in the tumors from CIRP/PyMT mice when compared to PyMT mice. This difference in immune cell cohorts is consistent with the mechanistic role of CIRP in decreasing cytokines associated with tumor progression in breast cancer [79,81,82].

Results suggest that CIRP may decrease breast tumor growth and metastases by decreasing these cytokines and changing the immune cell cohort present within these tumors. In particular, the decreased levels of IL-6, KC, MCP-1, VEGF-A, and CD4+ T cells in the tumors taken from 14-week-old CIRP/PyMT mice compared to PyMT mice could explain the difference in the number of pulmonary metastases observed between these two genotypes. No difference in the number of macrophages was observed between CIRP/PyMT mice and PyMT mice. However, it is possible that the macrophage polarity may be different, given the observed differences in cytokines. The question of whether the changed immune cell population resulted in or from the altered cytokines will be addressed in a future study.

Although a direct mechanism of how CIRP regulates inflammation has not been explored in our model, many studies have described how CIRP functions as a regulator of inflammation in both cancer and other diseases. Previous studies on CIRP as a modulator of inflammation in cancer have revealed that CIRP can act as a damage-associated molecular pattern (DAMP) and stimulate MyD88-NFκB and its subsequent cytokine release via Toll-like receptor 4 (TLR4) binding [23]. This mechanism of CIRP function was initially described in sepsis in both humans and murine models [16,81], and as a pro-tumorigenic event in both colorectal and liver cancer [18,19]. However, recent findings have shown that the ability of CIRP to function as a TLR4 ligand can also induce an antitumorigenic inflammatory response in mice with E.G7-OVA tumors, showing that this aspect of CIRP function can potentially be applied clinically to drive a tumor rejection response [82].

## 5. Conclusions

Our results, taken in context with previous findings, suggest that the role CIRP plays in inflammation in cancer is context-dependent, explaining why CIRP increases pro-tumorigenic inflammation in colorectal and liver cancers [18,19] while decreasing pro-tumorigenic inflammation in breast cancer, as well as decreasing inflammation in wound healing [70]. Future studies will focus on the mechanistic role CIRP plays in modulating cytokine levels to provide clarity on how CIRP variably modulates inflammation, including whether it requires an RNA-binding function. Further exploring how immune cell cohorts are affected, particularly regulatory T cells and macrophage polarization, within the tumor tissue and in blood will be key to determining CIRP’s function in human breast cancer.

## Figures and Tables

**Table 1:** Histopathology of mammary glands from 7-week-old CIRP/PyMT and PyMT mice.

	PyMT	CIRP/PyMT
**Primary Lesions**	Larger, less clearly demarcated	Smaller, more clearly demarcated
**Secondary Duct Involvement**	Considerably greater	Less secondary duct involvement
**Secondary Lesions**	More advanced	Less advanced
**Atypia**	No difference	
**Vascularity**		
**Inflammatory Cell Infiltrate**		
**Mean Mitotic Figures**	2.2 figures per field	0.7 figures per field
**Mean Apoptotic Figures**	1.15 figures per field	4.1 figures per field

## Data Availability

The data presented in this study are available within this article and the associated Supplementary Materials.

## Supplementary Materials

The following supporting information can be downloaded at https://www.mdpi.com/article/10.3390/biomedicines12020340/s1: Figure S1: CIRP knockdown efficiency; Figure S2: Human CIRP Protein Level in Serum; Table S1: Genotyping primers; Table S2: Description of antibodies; Table S3: Sequences of the Alt-R crRNAs (Guide RNAs); Table S4: Tissue supernatant assays of 14-week-old mammary tumors; Table S5: Serum cytokine from mice at 14 weeks of age.
