# Supplementary material for: Cold-Inducible RNA Binding Protein Impedes Breast Tumor Growth in the PyMT Murine Model for Breast Cancer
Source: Biomedicines. 2024 Feb 1;12(2):340. doi: 10.3390/biomedicines12020340 (PMC10886683; doi:10.3390/biomedicines12020340)
Supplement: Supplementary file 1 [file biomedicines-12-00340-s001.zip › biomedicines-2800627-supplementary.pdf]

Supplemental Figures and Tables

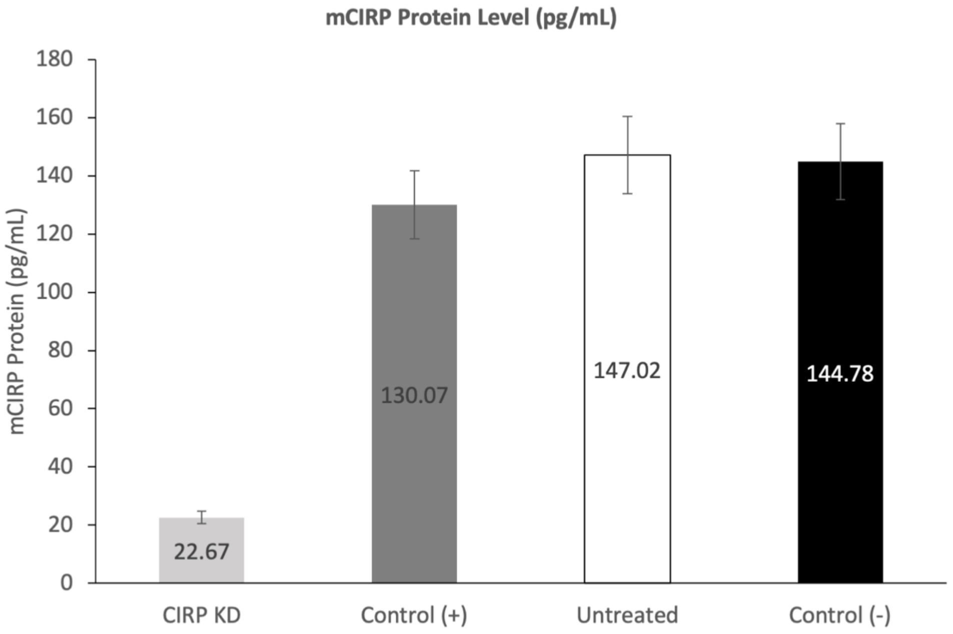

Figure S1. CIRP Knockdown Efficiency. Mouse CIRP protein was measured by ELISA and shown in picograms per milliliter. Cells were mixed with either positive (+) control (targeting the HPRT gene) crRNA RNP complex, negative (-) control crRNA RNP complex, CIRP crRNA RNP complex (CIRP Knockdown or KD), or lipofectamine only (untreated).

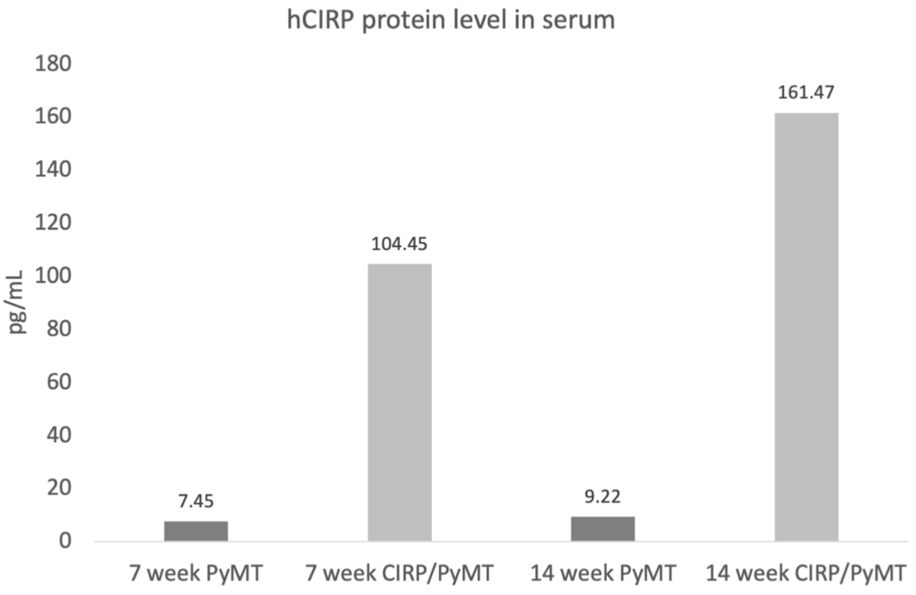

Figure S2. Human CIRP Protein Level in Serum. Human CIRP (hCIRP) protein in serum of the indicated mice (3-5 per genotype) was measured by ELISA and shown in picograms per milliliter.

Table S1: Genotyping Primers	
**Target Gene Name**	**Primer Sequence**
PyMT Forward	5′- CGG CGG AGC GAG GAA CTG AC 3′
PyMT Reverse	5′- TCA GAA GAC TCG GCA GTC TTA -3′
CIRP Forward	5′- TAC TAT AGC AGC CGG AGT CAG AGT -3′
CIRP Reverse	5′- AAC AGA TGG CTG GCA ACT AGA AGG -3′

Table S2: Description of Antibodies				
**Antibody**	**Source**	**Catalog Number**	**Dilution**	**Stock Concentration**
Ki67 (SP6)	NeoMarkers	RM-9106-S0	1:300	Not Provided
CD11b	Life Technologies	25-0112-82	0.1μg/10^6 cell per 100μl	0.2mg/ml
CD19	Life Technologies	47-0193-82	0.1μg/10^6 cell per 100μl	0.2mg/ml
F4/80	Life Technologies	61-4801-82	0.8μg/10^6 cell per 100μl	0.2mg/ml
CD8A	Life Technologies	53-0081-82	0.4μg/10^6 cell per 100μl	0.5mg/ml
Ly6G	Biolegend	127616	0.2μg/10^6 cell per 100μl	0.2mg/ml
Ly6C	Biolegend	128014	0.2μg/10^6 cell per 100μl	0.5mg/ml
CD3	Biolegend	100351	0.4μg/10^6 cell per 100μl	0.2mg/ml
CD4	Biolegend	100552	0.2μg/10^6 cell per 100μl	0.2mg/ml

Table S3: Sequences of Alt-R crRNAs (Guide RNAs)			
**Product**	**Sequence Name**	**Sequence**	**Number of Bases**
Alt-R CRISPR/Cas9 crRNA 10nmol	Mm.Cas9.CIRBP.1.AA	Alt-R1/UCU UCU GAC AAC CGG UCC CGG UUU UAG AGC UAU GCU/Alt-R2	36
Alt-R CRISPR/Cas9 crRNA 10nmol	Mm.Cas9.CIRBP.1.AB	Alt-R1/CAG CUU CGA CAC CAA CGA GCG UUU UAG AGC UAU GCU/Alt-R2	36
Alt-R CRISPR/Cas9 crRNA 10nmol	Mm.Cas9.CIRBP.1.AC	Alt-R1/AGC GUC AUC GAU AUU UUC AAG UUU UAG AGC UAU GCU/Alt-R2	36

Table S4: Tissue Supernatant Assays of 14-week Mammary Tumors		
Cytokines	PyMT	CIRP/PyMT
G-CSF	14364.084	12896.117
GM-CSF	702.420	541.183
IFNg	8.839	2.552
IL1a	109.398	125.054
IL1b	39.709	33.731
IL2	4.630	2.577
IL4	< 3.2	< 3.2
IL5	13.450	6.837
IL6	19232.755	10341.923
IL7	23.289	15.191
IL9	163.234	111.206
IL10	34.320	20.508
IL12 (p40)	< 3.2	< 3.2
IL12 (p70)	37.748	26.729
IL13	3.262	1.835
IL15	26.194	13.853
IL17	3.334	2.889
IP10	214.545	234.483
KC	8588.929	6032.330
MCP-1	3673.824	2405.679
MIP1a	180.150	114.504
MIP1b	177.447	106.123
MIP2	4119.141	3524.152
RANTES	48.557	41.849
TNFa	19.422	16.470

**Table S5: Serum Cytokines from Mice at 14-weeks of Age**		
Serum Cytokines	PyMT	CIRP/PyMT
G-CSF	1677.933	1756.418
GM-CSF	25.314	25.438
IFNg	< 3.2	< 3.2
IL1a	134.535	74.809
IL1b	19.730	11.038
IL2	2.423	1.144
IL4	< 3.2	< 3.2
IL5	12.502	6.419
IL6	7.302	7.087
IL7	12.976	6.909
IL9	32.310	60.814
IL10	2.309	5.893
IL12 (p40)	< 3.2	< 3.2
IL12 (p70)	7.217	3.992
IL13	< 3.2	< 3.2
IL15	5.255	7.450
IL17	1.550	0.840
IP10	117.829	99.076
KC	29.170	19.041
MCP-1	49.925	29.854
MIP1a	59.983	38.541
MIP1b	< 3.2	< 3.2
MIP2	64.914	30.046
RANTES	7.642	5.846
TNFa	5.881	4.472
